# Airborne Radioiodine: A Comparative View of Chemical Forms in Medicine, Nuclear Industry, and Fallout Scenarios

**DOI:** 10.3390/ijms27020590

**Published:** 2026-01-06

**Authors:** Klaus Schomäcker, Ferdinand Sudbrock, Thomas Fischer, Felix Dietlein, Markus Dietlein, Philipp Krapf, Alexander Drzezga

**Affiliations:** 1 Department of Nuclear Medicine, Faculty of Medicine and University Hospital Cologne, University of Cologne, Kerpener Str. 62, 50937 Cologne, Germany; ferdinand.sudbrock@uk-koeln.de (F.S.); thomas.fischer@uk-koeln.de (T.F.); markus.dietlein@uk-koeln.de (M.D.); philipp.krapf1@uk-koeln.de (P.K.); alexander.drzezga@uk-koeln.de (A.D.); 2 Computational Health Informatics Program, Boston Children’s Hospital, Harvard Medical School, Boston, MA 02115, USA; felix.dietlein@childrens.harvard.edu; 3 Forschungszentrum Jülich GmbH, Institute of Neuroscience and Medicine, Nuclear Chemistry (INM-5), Wilhelm-Johnen-Straße, 52428 Jülich, Germany; 4 German Center for Neurodegenerative Diseases (DZNE), Bonn-Cologne, Venusberg-Campus 1/99, 53127 Bonn, Germany

**Keywords:** airborne I-131, radioiodine therapy, routine reactor emissions, nuclear fallout, iodine speciation

## Abstract

Airborne iodine-131 plays a pivotal role in both nuclear medicine and nuclear safety due to its radiotoxicity, volatility, and affinity for the thyroid gland. Although the total exhaled activity after medical I-131 therapy is minimal, over 95% of this activity appears in volatile organic forms, which evade standard filtration and reflect metabolic pathways of iodine turnover. Our experimental work in patients and mice confirms the metabolic origin of these species, modulated by thyroidal function. In nuclear reactor environments, both under routine operation and during accidents, organic iodides such as [^131^I]CH_3_I have also been identified as major airborne components, often termed “penetrating iodine” due to their low adsorption to conventional filters. This review compares the molecular speciation, environmental persistence, and dosimetric impact of airborne I-131 across clinical, technical, and accidental release scenarios. While routine reactor emissions yield negligible doses (<0.1 µSv/year), severe nuclear incidents like Chernobyl and Fukushima have resulted in significant thyroid exposures. Doses from these events ranged from tens of millisieverts to several Sieverts, particularly in children. We argue that a deeper understanding of chemical forms is essential for effective risk assessment, filtration technology, and emergency preparedness. Iodine-131 exemplifies the dual nature of radioactive substances: in nuclear medicine its radiotoxicity is therapeutically harnessed, whereas in industrial or reactor contexts it represents an unwanted hazard. The same physicochemical properties that enable therapeutic efficacy also determine, in the event of uncontrolled release, the range, persistence, and the potential for unwanted radiotoxic exposure in the general population. In nuclear medicine, exhaled activity after radioiodine therapy is minute but largely organically bound, reflecting enzymatic and metabolic methylation processes. During normal reactor operation, airborne iodine levels are negligible and dominated by inorganic vapors efficiently captured by filtration systems. In contrast, major accidents released large fractions of volatile iodine, primarily as elemental [^131^I]I_2_ and organically bound iodine species like [^131^I]CH_3_I. The chemical nature of these compounds defined their atmospheric lifetime, transport distance, and deposition pattern, thereby governing the thyroid dose to exposed populations. Chemical speciation is the key determinant across all scenarios. Exhaled iodine in medicine is predominantly organic; routine reactor releases are negligible; severe accidents predominantly release elemental and organic iodine that drive environmental transport and exposure. Integrating these domains shows how chemical speciation governs volatility, mobility, and bioavailability. The novelty of this review lies not in introducing new iodine chemistry, but in the systematic comparative synthesis of airborne radioiodine speciation across medical therapy, routine nuclear operation, and severe accident scenarios, identifying chemical form as the unifying determinant of volatility, environmental transport, and dose.

## 1. Introduction

The radiation emitted by iodine-131 embodies both therapeutic potential and radiological risk. This radionuclide represents the archetype of a theranostic agent—a single isotope capable of both diagnosing and treating disease through its combined gamma and beta emissions [1,2]. In nuclear medicine, iodine-131 remains the classical model for targeted radiotherapy of differentiated thyroid carcinoma and, in selected oncologic indications, for the treatment of neuroendocrine tumors with [^131^I]-MIBG [3,4] or radioiodinated antibodies [5,6]. The same radiophysical properties that enable these therapeutic benefits also necessitate careful management, since iodine’s volatility and pronounced thyroid affinity can result in unintended exposures under medical or environmental conditions.

A critical but often underestimated aspect of radioiodine therapy (RIT) is the exhalation of volatile iodine species. These airborne emissions not only determine the potential radiation exposure of caregivers and bystanders but also provide insight into the biochemical pathways underlying iodine metabolism. A clear understanding of the nature and proportion of the emitted species—elemental, organically bound, and aerosol-associated iodine—helps to explain how iodine transitions between metabolic and environmental compartments [7,8].

Beyond medicine, airborne iodine-131 also plays an important role in the broader field of nuclear technology. During normal reactor operation, minor releases of gaseous iodine may occur, typically remaining well within authorized limits. In contrast, major nuclear accidents such as Chernobyl (1986) and Fukushima Daiichi (2011) resulted in uncontrolled, large-scale emissions of iodine-131 into the atmosphere, producing widespread yet heterogeneous exposure patterns. The speciation of radioiodine in these scenarios—its partitioning among molecular, organic, and particulate forms—was decisive for atmospheric transport, deposition, and ultimately thyroid dose [9]. Since iodine isotopes are accumulated almost exclusively in the thyroid gland, it is the critical organ-at-risk for radioiodine exposure. In Table 1 an overview on the dose factors for the different radioisotopes of iodine is included.

Interestingly, the oxidative transformations governing iodine speciation in such reactor environments are, in principle, analogous to those occurring within the human thyroid. In both settings, radiation and reactive oxygen species promote the conversion of iodide into volatile and/or organic compounds such as I_2_, HOI, or CH_3_I. Although one process unfolds in a damaged reactor core and the other within living tissue, both share the same underlying redox chemistry. Recognizing this analogy provides a unifying chemical perspective linking environmental and biological iodine pathways.

After the Chernobyl disaster, epidemiological studies documented a pronounced increase in childhood thyroid cancer, largely attributable to the inhalation and ingestion of iodine species with high bioavailability [10]. Although detailed speciation data remain limited, molecular iodine and organic compounds such as methyl iodide are recognized as particularly efficient in thyroidal uptake [11].

The objective of this review is to provide a comparative perspective on airborne iodine-131 species arising from medical applications, routine reactor emissions, and nuclear accidents. We examine how chemical speciation influences volatility, bioavailability, and dosimetric relevance, aiming to refine the understanding of exposure pathways and to strengthen the scientific foundation for radiological risk assessment.

While numerous studies and reviews have addressed iodine chemistry in medical applications, routine reactor operation, or severe nuclear accidents individually, these domains are typically treated in isolation. A systematic comparison across these contexts is largely lacking, despite the fact that the underlying chemical and radiolytic processes governing iodine speciation are closely related. The present review does not aim to introduce new iodine chemistry; rather, its novelty lies in the explicit comparative synthesis of airborne radioiodine behavior across medicine, nuclear industry, and accident scenarios. By integrating these perspectives, the review identifies chemical form as the unifying determinant of volatility, environmental transport, and dose, thereby providing a coherent framework that links clinical practice, nuclear safety, and radiological protection.

**Table 1:** Radiologically relevant iodine isotopes: physical properties, occurrence scenarios, and adult inhalation dose coefficients for elemental iodine vapor (I_2_) according to ICRP Publication 119 [12].

Isotope	Half-Life	Main Emissions	Typical Scenarios of Occurrence	Radiological Relevance	Notes/Justification	Inhalation Dose Coefficient (I_2_, Sv/Bq)
I-123	13.2 h	EC, γ	Diagnostic nuclear medicine	Diagnostic only	Short half-life, negligible environmental relevance	2.1 × 10^−10^
I-124	4.2 d	β^+^, γ	PET imaging	Diagnostic research	PET tracer, very low activities in clinic only	1.2 × 10^−8^
I-125	59.4 d	EC, Auger, γ	Brachytherapy seeds	Local therapy only	Low-energy emissions, not airborne relevant	1.4 × 10^−8^
I-126	13 d	EC, β^−^, β^+^, γ	Reactor fission product	Minor	Low cumulative yield, limited impact	2.6 × 10^−8^
I-129	16 My	β^−^	Reactor fission, reprocessing, fallout	Long-term tracer (radioecology), negligible acute dose	MBq-scale requires gram quantities in thyroid	9.6 × 10^−8^
I-130	12.4 h	β^−^, γ	Reactor accidents	Short-lived contributor	Relevant only immediately after release	1.9 × 10^−9^
I-131	8.0 d	β^−^, γ	Therapy, reactor releases, accidents	High contribution on collective doses Widely distributed into environment	Dominates internal dosimetry on a large scale	2.0 × 10^−8^
I-132	2.3 h	β^−^, γ	Reactor accidents	Relevant at early stages	Lower dose factor due the short half-live	3.1 × 10^−10^
I-133	20.8 h	β^−^, γ	Reactor accidents	Contributes significantly	Early-phase contributor, overshadowed by I-131	4.0 × 10^−9^
I-135	6.6 h	β^−^, γ	Reactor accidents or “iodine pit” in reactor control [13]	Relevant at early stages	Lower dose factor due the short half-live	9.2 × 10^−10^

## 2. Radioiodine Isotopes Relevant to Different Scenarios

To distinguish the isotopes of radiological concern across medical and environmental contexts, Table 1 summarizes their main physical characteristics and relevance to different exposure scenarios.

Among the numerous iodine isotopes generated in nuclear fission, only a subset is relevant for medical applications, environmental dispersion, and radiation protection. Table 1 summarizes isotopes occurring in nuclear medicine, during normal reactor operation, and under accident conditions. From a radiological perspective, I-131 remains the dominant contributor to thyroid dose over a wide area after accidental releases because of its intermediate half-life, high cumulative yield, and its high thyroid uptake fraction. Short-lived isotopes such as I-132, I-133, and I-135 may contribute substantially in the very early phase of a release but are of limited significance beyond a few days due to their rapid decay. By contrast, I-129, despite its extremely long half-life and relatively high per-Bq dose coefficient, is practically irrelevant for acute dose considerations: the mass required to achieve MBq activities in the thyroid is orders of magnitude higher than the gland’s physiological iodine capacity, making it a concern only for long-term environmental tracing and chronic low-dose exposure rather than for acute radiation protection. Among the medically applied isotopes, I-123 (produced mainly by cyclotron reactions on tellurium-124 or xenon-124), I-124 (cyclotron production from tellurium-124), and I-125 (reactor-based production via neutron capture on tellurium-124 possible) are of diagnostic or therapeutic use but play no role in environmental releases [14,15].

For these reasons, subsequent considerations will focus exclusively on the chemical forms and atmospheric behavior of I-131.

## 3. Chemical Form Matters: Mechanisms and Representative Reactions

The chemical behavior of airborne radioiodine (denoted as ‘I,’ with I-131 implied throughout) defies conventional textbook iodine chemistry. In medical, industrial, and reactor-accident environments, it is governed by a unique superposition of processes: aqueous redox reactions, radiolytic radical pathways, thermal decomposition, and heterogeneous surface reactions. Under severe accident conditions, this expands to include high-temperature gas-phase transformations and combustion-induced radical chemistry. Together, these coupled processes yield iodine species fundamentally different from those formed under ambient laboratory conditions.

Table 2A–D summarizes representative chemical transformation pathways of radioiodine across four distinct contexts:

(A) biological and mucosal environments relevant to medical radioiodine therapy,
(B) routine reactor operation and inspection conditions,
(C) Mo-99 production and spent-fuel reprocessing, and
(D) severe reactor accident scenarios involving radiolysis, high temperatures, structural degradation, and fires.

Each context is defined by a characteristic constellation of physicochemical stressors—including irradiated aqueous phases, intense γ-radiation fields, oxidizing or reducing conditions, organic matrices, metal and metal-oxide surfaces, and high-temperature vapor–solid equilibria—that govern the chemical form, stability, and potential volatilization of radioiodine species.

To maintain clarity and comparability, the reaction schemes are presented in a compact, scenario-specific format. Isotopic mass numbers are therefore not repeated within individual reactions; all pathways shown refer to radioiodine, predominantly I-131. Schematic notation is used where appropriate to represent experimentally established processes—such as surface-bound intermediates or radical chain reactions—that cannot be expressed as single elementary steps.

Notably, several reaction motifs—particularly radical-mediated oxidation, hydrolysis, and methylation processes—recur across multiple tables. This recurrence is intentional and reflects the fact that identical fundamental chemical mechanisms can operate under very different boundary conditions, while their relative relevance and observable outcomes differ between medical, industrial, and accident scenarios.

Taken together, Table 2A–D illustrates why radioiodine speciation, including the propensity for formation of volatile or airborne species, varies markedly between these contexts and why no single reaction pathway can account for the dominant species observed across all scenarios. The table series integrates insights from biological radiochemistry, reactor coolant chemistry, reprocessing science, and severe-accident research and provides a coherent conceptual anchor for the subsequent sections of this review.

While Table 2A–D outlines the four key scenarios in which airborne radioiodine may be generated—ranging from physiological processes in nuclear medicine to high-energy transformations in industrial and accidental settings—it also becomes evident that certain chemical reaction pathways recur across all of them. Despite their fundamentally different environments, these scenarios converge on a limited number of mechanistic routes that yield comparable volatile iodine species. For example, organically bound iodine emerges consistently, be it through enzymatic oxidation and subsequent methylation in the human body or through radiolytic and thermochemical pathways in reactor- or accident-related releases.

To make these cross-scenario parallels explicit, the following table highlights the core intersection points in chemistry, volatility generation, and exposure relevance. This integrative perspective clarifies that, although the origins differ profoundly, the reactive sequences governing airborne iodine speciation exhibit striking conceptual coherence.

With the mechanistic landscape now consolidated in Table 2A–D and Table 3, two conclusions become apparent. First, the chemical diversity of airborne iodine across the four scenarios is substantial, yet the underlying drivers—oxidation, hydrolysis, radiolysis, methylation, metal–iodine equilibria, and heterogeneous surface reactions—show a remarkable degree of convergence. Second, these pathways determine not only which species are formed but also their atmospheric persistence, volatility, and reactivity. In other words, chemistry defines the mobility of iodine in air.

Taken together, the reactions summarized in this section illustrate that iodine speciation is governed less by the origin of the system than by a limited set of recurring chemical drivers, including radiolysis, oxidation state cycling, surface interactions, and radical mediated methylation. The repeated appearance of these mechanisms across medical, technical, and accident related scenarios is therefore not coincidental but reflects shared underlying chemistry operating under different boundary conditions. Recognizing these common pathways provides a coherent framework for understanding why airborne iodine speciation differs in expression, yet remains mechanistically related, across the scenarios discussed in this review.

However, chemical form is only half of the story. Once airborne iodine is inhaled or deposited on moist surfaces, its physicochemical identity governs how efficiently it crosses biological interfaces, how rapidly it is converted to iodide, and how effectively it reaches the thyroid. The transition from environmental speciation to biological uptake therefore requires a shift in perspective—from reaction networks to biokinetics.

Section 4 builds on this foundation by examining how different airborne iodine species are incorporated into the human body, how they are transformed in physiological environments, and how their biokinetics determine thyroidal irradiation. This transition from atmospheric chemistry to internal dosimetry forms the conceptual bridge between environmental processes and their ultimate biological relevance.

## 4. Pathways of Incorporation and Systemic Distribution of Airborne [^131^I]Iodine

### 4.1. Transition from Physicochemical Form to Bioavailability

Radionuclides may enter the human body through several principal routes: inhalation of airborne materials, ingestion of contaminated food or water, percutaneous absorption through open wounds or damaged skin, and—rarely—transdermal diffusion across intact skin. Among these, inhalation represents the dominant pathway for airborne radioiodine released during reactor incidents, while ingestion becomes more relevant in the later environmental phase through contaminated milk, vegetables, and drinking water. Once incorporated, the physicochemical form—volatile, ionic, or particulate—determines its systemic availability and thyroid dose contribution [9,41].

Volatile compounds such as [^131^I]I_2_, [^131^I]HOI, and [^131^I]CH_3_I are absorbed across the pulmonary epithelium within minutes. In airway and blood plasma, they are rapidly reduced or hydrolyzed to free iodide ([^131^I]^−^), which is fully bioavailable and actively accumulated in the thyroid via the sodium–iodide symporter (NIS) [42,43]. These species differ markedly in volatility and atmospheric persistence: while [^131^I]CH_3_I is highly volatile and capable of long-range atmospheric transport, [^131^I]I_2_ and particularly [^131^I]HOI are more reactive and prone to rapid removal by hydrolysis or wet deposition. [^131^I]HOI is not released directly but forms in moist, oxidizing air through photochemical or radiolytic reactions of molecular iodine with water or hydroxyl radicals and is rapidly converted back to iodide in aqueous environments.

Soluble aerosol salts like [^131^I]CsI dissolve readily in the airway lining fluid, releasing ionic [^131^I]^−^, which is efficiently absorbed into the bloodstream and taken up by the thyroid. In contrast, some airborne radioiodine can become incorporated into or adsorbed onto particulate matter of varying solubility. As outlined in ICRP Publications 60 and 119, pulmonary clearance of inhaled materials is governed primarily by their dissolution rate in lung fluids. These materials are categorized as absorption type F (fast), M (moderate), or S (slow) [12,44].

Rapidly dissolving (Type F) species are cleared within hours to days. Sparingly soluble or refractory (Type S) aerosols, however, may persist in the alveolar region for weeks to months before macrophage-mediated removal. Aerosols in which iodine is associated with mineral or metallic matrices may initially behave as Type S materials. Such particles contribute primarily to a localized pulmonary dose rather than to systemic irradiation. This biokinetic framework adequately encompasses the spectrum of airborne iodine forms encountered in reactor incidents without the need to specify individual chemical compounds.

Although truly insoluble iodine compounds are thought to contribute only marginally to systemic thyroid exposure, mixed aerosols containing iodine associated with mineral or metallic matrices can transiently behave as “slow” materials. Such particles contribute mainly to localized pulmonary dose rather than to systemic irradiation. This biokinetic framework adequately encompasses the spectrum of airborne iodine forms encountered in reactor incidents without the need to specify individual chemical compounds.

As summarized in Table 2D, iodine oxides (IO_x_) may form in oxidizing containment atmospheres. After deposition in biological environments, these oxides may undergo hydrolytic or reductive transformation to iodide (I^−^) in aqueous environmental films or aerosol particles, as indicated by recent environmental studies on IO_x_ chemistry [45,46]:

[^131^I] IO_x_ → [^131^I]^−^ + x H_2_O

Organically bound iodine compounds such as [^131^I]CH_3_I are highly volatile and lipophilic, allowing rapid diffusion through alveolar membranes into the bloodstream. In plasma and tissues, these molecules undergo enzymatic or spontaneous hydrolysis, releasing free [^131^I]^−^ and thereby restoring systemic bioavailability. This demethylation process is catalyzed by hepatic microsomal enzymes and nonspecific esterases, which convert volatile organoiodine species into inorganic iodide available for thyroidal uptake. Thus, even exhaled organic iodine indirectly contributes to systemic dose through subsequent metabolic degradation to free iodide [47,48,49].

Overall, the bioavailability of airborne [^131^I] is governed by its volatility, solubility, and redox stability. Volatile and soluble species yield immediate systemic distribution with predominant thyroid uptake, whereas insoluble metal iodides and oxide nanoparticles remain largely confined to pulmonary or reticuloendothelial compartments. Organically bound iodine compounds such as [^131^I]CH_3_I, while less reactive, are readily absorbed and undergo rapid hepatic glutathione-mediated degradation to free [^131^I]I^−^, ensuring eventual systemic availability. Accordingly, near-field exposures after reactor accidents are dominated by particulate [^131^I]-iodides, while organoiodine vapors prevail in the far-field environment.

### 4.2. Thyroidal Iodine Organification

Once [^131^I]^−^ is circulating in the bloodstream, it is actively transported across the basolateral membrane of thyroid follicular cells via the sodium–iodide symporter (NIS), which cotransports two sodium ions for each iodide ion under regulation by thyroid-stimulating hormone (TSH). Inside the thyrocyte, iodide diffuses toward the apical membrane, where it undergoes oxidation catalyzed by thyroid peroxidase (TPO). This enzymatic process requires hydrogen peroxide (H_2_O_2_) generated locally by dual oxidase 2 (DUOX2) [50].

The oxidized or “activated” iodine immediately reacts with tyrosine residues of thyroglobulin (Tg) to form monoiodotyrosine (MIT) and diiodotyrosine (DIT). In a subsequent coupling step, also mediated by TPO, two iodotyrosine residues are joined: MIT with DIT yields triiodothyronine (T_3_), and DIT with DIT yields thyroxine (T_4_) [42,51].

These iodinated forms of Tg remain stored within the follicular colloid until TSH stimulation triggers their endocytosis and proteolytic cleavage, releasing T_3_ and T_4_ into the bloodstream. Both oxidation and organification can be transiently inhibited by goitrogenic substances such as thiocyanate, perchlorate, or by the Wolff–Chaikoff effect, which temporarily suppresses iodide uptake or its incorporation into Tg under conditions of high iodine exposure [52,53].

Although iodide organification in the thyroid represents the principal pathway of iodine fixation in the human body, the volatile organoiodine fraction detected in exhaled air is unlikely to originate directly from thyroglobulin-bound iodine. Once incorporated into monoiodotyrosine (MIT), diiodotyrosine (DIT), or thyroid hormones (T_3_, T_4_), iodine remains covalently bound and is released into circulation only after proteolytic degradation of thyroglobulin.

In contrast, the organically bound ^131^I fraction observed in exhaled air—accounting for more than 70% of total exhaled activity both in our murine experiments and in clinical studies after radioiodine therapy—is more likely derived from peripheral metabolic or oxidative methylation processes [7,54].

These pathways generate low-molecular-weight volatile iodine compounds such as [^131^I]CH_3_I, which readily diffuse across the pulmonary epithelium.

The marked reduction in exhaled organic ^131^I activity following pharmacologic inhibition of thyroid function (e.g., by perchlorate, carbimazole, or L-thyroxine pretreatment) suggests, however, that thyroidal iodine turnover indirectly contributes to the systemic iodine pool available for volatile compound formation. Thus, the thyroid itself does not emit volatile iodine species but modulates their systemic availability through its role in iodine metabolism and turnover.

### 4.3. Dosimetric and Radiobiological Aspects of ^131^I Uptake in the Thyroid

After systemic absorption, [^131^I]^−^ is rapidly concentrated in the thyroid and incorporated into thyroglobulin (Tg) within the follicular lumen. The intrathyroidal retention of radioiodine results in localized β^−^ irradiation of the follicular epithelium and colloid. The β^−^ particles emitted by I-131, with a mean energy of approximately 190 keV and an average tissue range below 1 mm, account for nearly the entire therapeutic effect. By contrast, the accompanying 364-keV γ-emission contributes little to the thyroidal absorbed dose but dominates external and whole-body exposure components [55,56,57].

The absorbed dose to the thyroid is determined by the cumulated activity within the gland (Ã_thy) and the corresponding S-value, representing energy deposition per unit activity. For a typical adult thyroid (~20 g) with a 25% uptake fraction and an effective half-life of about 8 days, the absorbed dose is approximately 0.4 Gy per MBq administered [58], consistent with empirical dosimetric data from radioiodine therapy of benign thyroid disorders. This estimate aligns with empirical dosimetric data obtained in radioiodine therapy for benign thyroid disorders.

The effective dose delivered to the thyroid is governed not only by the cumulated glandular activity but also by the physicochemical form and route of intake of ^131^I. Different compounds and absorption types exhibit marked variability in systemic availability and resulting committed effective dose per unit intake.

Table 4 summarizes the effective dose coefficients (Sv Bq^−1^) for relevant iodine species and absorption classes, as provided by ICRP Publication 119 [12], to illustrate how volatility, solubility, and chemical reactivity influence thyroidal exposure.

Adult ICRP reference values are shown; pediatric and pregnancy-specific coefficients are available in the underlying ICRP models but are beyond the scope of this summary table.

#### Cellular Dosimetry

At the cellular level, β-emission from I-131 causes both direct and indirect DNA damage [59,60]. Studies in glioblastoma and thyroid or lymphocyte cell models show induction of single- and double-strand breaks, chromosomal aberrations, and oxidative base damage [61,62]. In cancer cell lines, I-131 has been shown to induce apoptosis via downregulation of antiapoptotic genes such as BCL-2 and BCL-XL, indicating activation of intrinsic apoptotic pathways [63]. Furthermore, oxidant stress from reactive oxygen species (ROS) may impair DNA repair kinetics, exacerbate genomic instability, or trigger senescence pathways. These subcellular injuries form the mechanistic basis for the macroscopic thyroidal damage observed in therapy and exposure settings [64].

The physicochemical form primarily determines how rapidly radioiodine becomes systemically available as iodide (I^−^). Volatile species such as [^131^I]I_2_, [^131^I]HOI, and [^131^I]CH_3_I are efficiently absorbed across the pulmonary epithelium, reduced to iodide, and subsequently accumulated in the thyroid. In contrast, particulate forms of varying solubility—classified by the ICRP as absorption types F (fast), M (moderate), or S (slow)—exhibit longer residence times in the respiratory tract [12]. Their gradual dissolution delays systemic transfer, thereby diminishing the immediate thyroid dose but increasing localized pulmonary irradiation. Ingested iodide and molecular iodine are rapidly absorbed through the gastrointestinal tract, yielding high thyroidal uptake, particularly in children, whose thyroid avidity for iodide exceeds that of adults by several fold [42]. Percutaneous or wound contamination, though rare, is radiobiologically equivalent to direct systemic uptake once iodide reaches circulation [65].

Therapeutically, the β-radiation emitted by I-131 induces cellular damage and apoptosis in thyroid follicular cells, resulting in volume reduction or ablation of hyperfunctioning tissue—an effect exploited in the treatment of Graves’ disease, toxic multinodular goiter, and differentiated thyroid carcinoma. At higher absorbed doses, nearly complete ablation of the thyroid is achieved, whereas at lower doses, partial cytoreduction occurs without destruction of the gland’s architecture [66].

The deterministic effects of ^131^I exposure are confined to thyroid tissue, whereas stochastic risks are mainly associated with radiation-induced carcinogenesis. Epidemiological evidence, notably from post-Chernobyl cohort studies, demonstrates a clear dose–response relationship between childhood thyroidal ^131^I exposure and subsequent papillary thyroid carcinoma, with risk most pronounced in those youngest at exposure.

In contrast, data for exposures during adulthood are less definitive but generally indicate a substantially lower and often statistically inconclusive excess risk [67,68].

Protective interventions such as pre-exposure administration of stable iodine (KI) effectively reduce thyroid dose by competitively inhibiting NIS-mediated uptake, whereas elevated TSH levels enhance radioiodine accumulation—a mechanism deliberately utilized in therapeutic protocols. Conversely, goitrogenic substances and the transient Wolff–Chaikoff effect may suppress iodine uptake or organification under high iodine load, thereby modifying thyroidal retention kinetics [69].

## 5. Airborne ^131^I in Practice: Nuclear Medicine, Routine Reactor Operation, and Accidents

### 5.1. Nuclear Medicine (Post-Therapy Exhalation)

Following oral administration of therapeutic radioiodine, only a minute fraction of the applied activity is released into room air via exhalation. Quantitative investigations conducted at our institution demonstrated that approximately 0.01–0.03% of the administered I-131 activity is exhaled, predominantly in organically bound form (>90%), such as I-131-methyl iodide, with the remainder occurring as molecular iodine or particulate iodides [7,54,70]

Because of their high volatility and chemical stability, these organically bound iodine species can pass conventional ventilation filters largely unretained; however, they disperse rapidly once released into ambient air. Consequently, measurable external or inhalation doses to other individuals occur only in close proximity to the patient and mainly during the first few hours after administration.

Although only about 0.01–0.03% of the administered activity is exhaled, this fraction is released predominantly as organic iodine species, which are less efficiently captured than I_2_ or HOI. The practical relevance of this pathway has been quantified by Sudbrock et al., who modeled domestic exposure scenarios based on our previously published exhalation data [8]. Their results showed that—even under worst-case assumptions—measurable but sub-millisievert thyroid doses may occur only during close personal contact within the first hours after therapy. In routine practice, however, inhalation contributes only a minor share of overall exposure, with body-fluid contamination remaining the dominant pathway.

These findings primarily provide biochemical and mechanistic insight into iodine metabolism at the individual level and should not be interpreted as indicating a relevant contribution to public radiation exposure.

Accordingly, the standard precautions already included in routine patient information—adequate ventilation, avoiding prolonged face-to-face contact with children or pregnant individuals, and separate sleeping arrangements for one night—are sufficient to limit exposure to levels far below regulatory concern.

### 5.2. Routine Operation of Nuclear Power Plants

During routine reactor operation, airborne concentrations of I-131 are extraordinarily low and, for several decades, have shown a pronounced downward trend.

Early environmental monitoring campaigns conducted in the 1970s and early 1980s occasionally detected measurable I-131 activities in the near-field of reactor sites—typically within a few hundred meters of the ventilation stack—at concentrations on the order of 10^−3^ to 10^−2^ Bq m^−3^ [71,72].

In contrast, continuous surveillance networks in Europe and Japan now report values consistently below the detection limit of high-purity germanium spectrometers, corresponding to about 10^−6^ Bq m^−3^, despite sampling air volumes of several hundred cubic meters.

This three- to four-order-of-magnitude decrease over the past four decades [73,74,75] reflects major technical improvements in fuel-rod tightness, primary-circuit purification, off-gas filtration, and containment design, leading to an almost complete elimination of airborne iodine releases.

The chemical speciation of residual iodine near plant stacks remains dominated by molecular iodine ([^131^I]I_2_) and hypoiodous acid ([^131^I]HOI), species that can be detected only in close proximity to the exhaust system [20,76,77].

Trace formation of volatile organic iodides such as [^131^I]CH_3_I is theoretically possible through radiolytic or catalytic methylation of iodide in moist, oxidizing atmospheres; however, their actual concentrations in reactor off-gas are below analytical significance [35,78].

Although [^131^I]CH_3_I is intrinsically more challenging to remove by conventional activated-charcoal or silver-impregnated filtration systems than [^131^I]I_2_ or [^131^I]HOI, its contribution to the total iodine-131 inventory in stack effluents remains negligible under normal operating conditions [25,79,80].

Measurements at these sensitivity levels are typically performed within established radiological environmental monitoring programs (REMP), which include air-sampling stations located near the plant boundary—usually within one kilometer of the main stack—and additional control sites at 15–30 km distance in the upwind direction [81].

Current high-volume sampling with charcoal/TEDA cartridges followed by low-background HPGe γ-spectrometry yields typical detection limits for gaseous ^131^I of ~6 × 10^−5^ to 3 × 10^−4^ Bq m^−3^, with specialized long-integration setups reaching the µBq m^−3^ range; thus, the present absence of measurable airborne iodine reflects genuinely low releases rather than analytical insensitivity [82,83].

Accordingly, the total annual public dose from all airborne radionuclides near modern nuclear power plants is typically below 0.1 µSv y^−1^, with ^131^I accounting for less than one percent of that value [84,85].

### 5.3. Reactor Shutdown and Inspection

During planned or unplanned shutdowns of light-water reactors and subsequent inspection phases, minor releases of radioiodine may occur as a result of pressure and temperature transients in the reactor coolant system and fuel-clad gaps. Studies of “iodine-spiking” phenomena show that when coolant enters the pellet-clad gap during cooldown, previously deposited water-soluble iodine may be mobilized and released into the primary circuit, particularly as the cladding cools and condensation occurs [86].

The magnitude of such releases is typically orders of magnitude lower than during full core damage or accident scenarios; nevertheless, the released species often include iodide-131 ([^131^I]I^−^) in aqueous form as well as volatile molecular iodine ([^131^I]I_2_) or organic iodides, whose retention depends on gap chemistry and the efficiency of available scrubbing processes [87]. Because the majority of the iodine-131 inventory decays quickly once fission is terminated, any release during inspection intervals is typically small and confined to internal system boundaries rather than to the external environment [88].

### 5.4. Reprocessing Facilities and Mo-99 Production

Airborne releases of radioactive iodine also occur in industrial facilities that handle fission products during fuel reprocessing or medical isotope production. In both contexts, iodine-131 is generated by the fission of uranium-235 and may be released as volatile species during dissolution or chemical separation of irradiated targets.

In nuclear fuel reprocessing plants, dissolution of spent fuel in nitric acid partitions iodine into the off-gas stream, where volatile species—primarily [^131^I]I_2_, with radiolytic formation of [^131^I]CH_3_I—are expected. Multistage off-gas treatment systems, typically combining silver-zeolite and activated carbon, achieve overall decontamination factors exceeding 10^5^ [21].

Representative regulatory inventories indicate that routine gaseous ^131^I discharges at UK nuclear sites are in the range of 10^7^–10^8^ Bq y^−1^, i.e., well below the reporting threshold of 10^9^ Bq y^−1^ [89,90].

For La Hague and Sellafield, national oversight reports attribute gaseous iodine effluents mainly to shearing and dissolution steps; these streams are efficiently treated prior to release, resulting in very low stack activities under normal operation [91].

In medical isotope production facilities—particularly those manufacturing molybdenum-99 from uranium fission—volatile iodine-131 is released during target dissolution and radiochemical separation steps. Despite the much smaller throughputs compared to reprocessing plants, Mo-99 production can constitute a significant localized source of airborne iodine because I-131 is co-produced in measurable quantities. Stack monitoring at such facilities (e.g., ANSTO, Australia; NTP, South Africa; Mallinckrodt, Netherlands) consistently shows that releases are dominated by organic iodine species, chiefly methyl iodide and related compounds, formed through oxidative and radiolytic methylation in nitric acid solutions [28,92,93,94].

Absolute ^131^I discharges are typically small, on the order of <10^−8^ Bq per batch; nevertheless, efficient capture systems and continuous monitoring remain necessary, as organoiodine vapors exhibit higher penetration through conventional filtration systems than molecular iodine. Advances in silver-zeolite and molecular-sieve technology have reduced such emissions to levels corresponding to annual public doses well below 0.1 µSv y^−1^ [93].

### 5.5. Severe Reactor Accidents

While numerous reactor incidents have occurred since the dawn of the nuclear age, only four are known to have released radioiodine to an extent relevant for public health and environmental contamination—Windscale (1957), Three Mile Island (1979), Chernobyl (1986), and Fukushima Daiichi (2011). Each event differed markedly in reactor type, containment integrity, and the chemical forms of iodine released.

#### 5.5.1. Windscale Fire (Sellafield, 1957)

In October 1957 a fire broke out in the graphite-moderated air-cooled reactor “Pile 1” at the Windscale site (now part of Sellafield) in north-west England. The incident led to the release of significant amounts of radioactive iodine-131: recent estimates suggest up to 1 800 TBq were emitted [95]. Unlike a typical meltdown release, the fire and uranium oxidation produced volatile iodine in elemental (I_2_) and possibly organic forms (e.g., CH_3_I), which disseminated into the atmosphere and deposited on pasture and milk [96]. Monitoring of milk in the surrounding 200 sq mile area showed elevated I-131 levels, prompting destruction of about one month’s milk production and thereby significantly reducing thyroid exposures.

Although individual thyroid doses in children reached tens of milligray (with some exceeding 100 mGy), long-term epidemiological studies up to 2020 did not demonstrate a statistically significant increase in thyroid cancer incidence in the most exposed cohort [95].

#### 5.5.2. Three Mile Island, 1979 (Pennsylvania, USA)

The accident at the Three Mile Island Unit 2 (TMI-2) pressurized-water reactor on 28 March 1979 was triggered by a loss-of-coolant event followed by operator errors and malfunctioning instrumentation. The partial core meltdown led to a release of noble gases and fission products, including radioiodine, from the damaged fuel rods into the containment atmosphere.

The total I-131 release to the environment has been estimated at approximately 15 Ci (≈0.55 TBq), representing less than 10^−8^ of the total iodine inventory of the core (about 2.3 PBq). Within the containment, most iodine existed as molecular iodine (I_2_) and aerosol-bound iodides, with a minor fraction of organic iodides (CH_3_I and higher alkyl iodides) formed through radiolytic methylation of iodine in the humid, carbon-bearing containment atmosphere [76,97,98,99,100,101].

Releases to the environment occurred primarily through filtered venting of the containment building; the chemical species of iodine reaching the atmosphere were therefore largely oxidized or particulate, with only traces of volatile organic forms [102]. Atmospheric dispersion modeling and environmental sampling confirmed deposition of I-131 at levels of only a few × 10^−3^ Bq m^−3^ in the immediate vicinity of the plant [98].

The resulting thyroid doses to the most exposed individuals within 10 km of the plant were estimated at 0.07–2 mSv, with an average population dose below 0.02 mSv, several orders of magnitude lower than natural annual background exposure. Epidemiological follow-up studies extending over four decades have shown no statistically significant increase in thyroid cancer or other radiation-related health effects in surrounding communities [103].

Environmental monitoring during and after the event did not detect measurable radioiodine above background beyond the immediate vicinity of the reactor. Consequently, the Three Mile Island accident—while a critical turning point in nuclear safety—represents a case where the chemical speciation of released iodine was dominated by non-volatile forms and the radiological impact from I-131 remained negligible compared with subsequent major reactor accidents.

#### 5.5.3. Chernobyl Accident (26 April 1986)

Source term and timing:

Unit 4 at the Chernobyl Nuclear Power Plant released a very large inventory of fission products to the atmosphere. Best-estimate releases exclude noble gases and place I-131 at ~1.7–2.0 EBq, corresponding to ~50–60% of the core inventory (~3200 PBq); Cs-137 releases were ~85 PBq (20–40%). The release persisted for more than a week with two pronounced peaks: an initial, mechanically driven burst in the first days (fuel fragmentation) and a second peak around days 7–10 associated with high core temperatures; subsequently, releases fell sharply as the core cooled [104,105,106].

Chemical and physical forms (speciation):

Material emitted to air comprised gases, aerosols and finely fragmented fuel. For iodine specifically, elemental iodine (I_2_), organic iodides (notably methyl iodide, CH_3_I), and aerosol-/oxidation products were present; organically bound iodine was explicitly detected in the plume, and the ratios among iodine compounds varied over time. Quantitatively resolved, time-continuous speciation for the whole release is not available; however, severe-accident source-term assessments for Chernobyl indicate that a large volatile fraction was involved, consistent with the observed long-range transport and vegetation interception. In-containment/atmospheric chemistry further supports rapid conversion of I_2_/organic iodides to HOI and iodine oxides (I_x_O_γ_), which can nucleate iodine-oxide aerosols and modify deposition efficiencies downwind [104,107,108,109,110,111].

Dose and pathways:

In the most affected regions (Belarus, northern Ukraine, western Russia), children’s thyroid doses from I-131 typically ranged ~0.5–3 Gy, with local maxima > 10 Gy where fresh milk consumption was high; adult thyroid doses were usually <0.2 Gy, and effective whole-body doses to the general population were commonly ~10–50 mSv. The dominant pathway was ingestion (milk, leafy greens) after pasture interception; inhalation contributed mainly during plume passage. Liquidators with measured thyroid burdens had mean doses around 0.18 Gy (median 0.11 Gy), with some up to ~4–5 Gy [9,41,112].

Health impacts:

A clear, dose-related increase in childhood/adolescent papillary thyroid carcinoma was documented in Belarus and Ukraine, with thousands of cases emerging in the 1990s; robust dose–response has been demonstrated, while no consistent excess of other solid cancers or leukemia attributable to I-131 has been established to date. Molecular analyses frequently show rearrangements typical of radiation-associated PTC [10,113,114]

Countermeasures and lessons:

Evacuation, dietary controls (especially milk), and stable-iodine (KI) prophylaxis (Poland!) where promptly applied reduced thyroid uptake significantly; timing is critical for efficacy. The event underscores how speciation [^131^I]I_2_/[^131^I]CH_3_I vs. particulate) governs transport, interception and ultimately thyroid dose, particularly in iodine-deficient populations with high milk consumption [115,116,117].

#### 5.5.4. Fukushima Daiichi Nuclear Accident (2011)

The Fukushima Daiichi nuclear accident in March 2011, triggered by the Great East Japan Earthquake and the subsequent tsunami, represented the most serious nuclear event since Chernobyl. Yet in reactor design, release mechanisms, and public health impact, the two accidents differed profoundly. Whereas the RBMK-1000 reactor in Chernobyl had no pressure vessel and no true containment, the Fukushima boiling-water reactors (Units 1–3) were equipped with reinforced containment structures. These barriers, together with prompt emergency responses, decisively limited the magnitude and biological consequences of radionuclide release [118,119,120].

Release and Chemical Forms of Radioiodine:

The total release of [^131^I] from the Fukushima Daiichi site has been estimated at approximately 150–200 PBq—roughly one-tenth of the activity emitted during the Chernobyl disaster. While multiple fission products were dispersed, iodine isotopes dominated the early internal exposure pathways. In the immediate aftermath, the atmospheric iodine consisted largely of molecular [^131^I]I_2_, volatile organic iodides such as [^131^I]CH_3_I, and, to a lesser extent, aerosol-associated iodine species. Their relative proportions depended on temperature, humidity, and reactor conditions, reflecting the complex thermochemistry of iodine volatilization from damaged fuel and coolant systems [121,122,123].

Unlike Chernobyl, where most radioiodine entered the food chain through contaminated pasture and milk, human exposure in Japan was dominated by short-term inhalation and limited ingestion before stringent countermeasures were implemented. Rapid interdiction of milk and leafy vegetables, coupled with evacuation of nearby populations, substantially reduced thyroid doses [124].

Thyroid Dose and Internal Exposure:

UNSCEAR and national monitoring programs concluded that the average committed equivalent thyroid doses among residents near Fukushima were generally below 10 mSv, often closer to 1 mSv. These represent organ-specific internal doses from inhaled or ingested radioiodine, not whole-body effective doses. Only small subgroups—emergency workers or individuals remaining within 20 km during the early phase—may have received several tens of millisieverts. These values are 100- to 1000-fold lower than the thyroid doses in children from heavily contaminated Chernobyl regions, which often exceeded 0.5–1 Gy [118,125,126].

The comparatively low exposures reflect both the containment integrity and the rapid implementation of protective measures, including sheltering, evacuation, food control, and, in selected cases, prophylactic administration of stable iodine (KI). Although the latter was not uniformly applied, it effectively reduced thyroidal uptake of [^131^I] in risk groups [127].

Health Implications:

A comprehensive thyroid-screening program involving more than 300 000 residents of Fukushima Prefecture revealed an apparent increase in diagnosed papillary thyroid carcinomas over the subsequent decade. However, detailed analyses indicate that this rise primarily reflects enhanced diagnostic surveillance rather than radiation causation. No statistically significant correlation has been established between individual thyroid doses and cancer incidence.

In stark contrast to the dose-related surge in childhood thyroid carcinoma following Chernobyl, Fukushima demonstrates how modern containment, rapid communication, and timely countermeasures can drastically mitigate the biological sequelae of large-scale radionuclide release. To date, no radiation-related increase in non-thyroid malignancies, congenital defects, or heritable effects has been demonstrated in the affected population [128,129].

Environmental Transport and Deposition:

Atmospheric-dispersion models indicate that a large fraction of released [^131^I] was transported eastward over the Pacific Ocean, leading to significant dilution and marine deposition rather than extensive terrestrial contamination. This oceanic sink reduced the long-term ingestion pathway that had dominated the Chernobyl scenario. Ground deposition in Japan was spatially heterogeneous, governed by rainfall patterns during the plume passage between 12 and 16 March 2011 [121].

Comparative Perspective

Fukushima thus stands as a counterpoint to Chernobyl: a modern nuclear accident in which comparable radionuclides were mobilized, yet their biological impact remained orders of magnitude lower owing to engineered containment and effective emergency management. Both events reaffirm that the speciation of radioiodine—its partitioning between elemental, organic, and particulate forms—governs atmospheric transport, environmental interception, and ultimately thyroid dose in human populations. The lesson is as chemical as it is radiological: the fate of iodine, once released, depends less on its quantity than on its molecular form and the promptness of human response.

## 6. Chemical Form Matters

The comparative analysis of medical and environmental data underscores a unifying principle: the chemical form of iodine determines its fate in both living systems and the atmosphere. This becomes most evident in large-scale events such as the Chernobyl accident, where the speciation of radioiodine decisively governed its atmospheric transport and biological impact. In contrast to particulate cesium isotopes, a major fraction of iodine was emitted in gaseous form. Early field measurements and modeling indicate that roughly three quarters of the total [^131^I] activity escaped as volatile species, while the remainder was aerosol-bound. Within this gaseous fraction, elemental iodine (I_2_) and methyl iodide (CH_3_I) predominated, their relative proportions changing with time and atmospheric oxidation state. Analyses of air filters across Europe revealed a growing organic fraction as the plume aged, consistent with photochemical methylation and oxidation in the troposphere.

Volatility largely determines atmospheric residence and range. Methyl iodide, with an atmospheric lifetime of several days, can travel hundreds to more than a thousand kilometers, whereas I_2_ and aerosol-bound iodine deposit efficiently within the near field. Experimental and environmental observations alike show that organically bound iodine represents the dominant volatile form, arising from chemical and radiolytic methylation processes. This mechanism explains why radioiodine released from reactor accidents or medical sources can persist in chemically stable, low-reactivity forms [130,131].

Particle size further modulates exposure relevance. After Chernobyl, airborne iodine- and cesium-bearing particles typically had submicron diameters that allowed them to remain suspended for days while still depositing efficiently near the source. Consequently, aerosol-bound iodine dominated local deposition and ingestion pathways, contaminating vegetation and milk, whereas volatile organic iodine—chiefly CH_3_I—governed regional to intercontinental dispersion [132].

Taken together, these observations delineate a clear scale separation: particulate iodine defines near-source dose and food-chain transfer, while volatile organic iodine determines the large-scale footprint of contamination. The chemical form thus links physics, chemistry, and biology—it dictates how far iodine travels, how long it persists, and how readily it becomes bioavailable in the environment.

## 7. Conclusions

Radioiodine embodies the dual nature of modern nuclear science: it is an essential tool for effective and often life-saving medical therapy, while simultaneously representing a persistent challenge for nuclear safety and radiological protection. Across all contexts considered in this review, the chemical form of radioiodine emerges as a key determinant for its volatility, environmental transport, and retention.

In nuclear medicine, patient exposure is dominated by radiation emitted from within the body, whereas airborne radioiodine released by patients represents only a minor and highly context-dependent pathway that may be relevant primarily for the potential incorporation of third parties. In contrast, in nuclear facilities and accident scenarios, the presence of volatile inorganic and organic iodine species directly affects containment performance, atmospheric dispersion, and environmental contamination. In severe accidents, iodine speciation has therefore played a decisive role in shaping population exposure and public-health consequences.

This chemical dependence is not coincidental. The same radiolytic and oxidative processes that influence iodine behavior under reactor conditions also operate in biological systems such as the thyroid, albeit at vastly different scales and under different boundary conditions. In both technological and biological environments, these processes govern the transformation of iodide into more volatile or organic forms.

Ultimately, iodine speciation provides a conceptual bridge between physics, chemistry, and biology. It determines how radioiodine is released, transported, and incorporated, and thus how exposure scenarios arise. Effective protection and the responsible use of radioiodine therefore require a shift from an exclusive focus on total activity toward a chemically informed understanding of iodine behavior. In this sense, managing the consequences of iodine-131 means understanding and controlling its chemical form.

Beyond summarizing established findings, this review brings together medical, industrial, and accident-related iodine chemistry within a single comparative framework. By consistently emphasizing chemical speciation while clearly distinguishing between exposure pathways, it aims to support a more integrated and chemically sound assessment of radioiodine-related risks across disciplines.

## Figures and Tables

**Table 2:** (**A**). Representative chemical pathways of radioiodine (^131^I) in the medical context (radioiodine therapy, exhalation) [16,17,18,19]. (**B**). Representative chemical pathways of radioiodine (^131^I) during routine reactor operation and inspection [20,21,22,23,24,25]. (**C**). Representative chemical pathways of radioiodine (^131^I) during reprocessing and Mo-99 production [21,26,27,28,29,30,31]. (**D**). Representative chemical pathways of radioiodine (^131^I) under severe reactor accident conditions [21,32,33,34,35,36,37,38,39,40,41].

Scenario/Reaction	Reaction	Explanation (Compact)
(**A**)		
A1: Enzymatic peroxidase oxidation	H_2_O_2_ + 2 I^−^ + 2 H^+^ → I_2_ + 2 H_2_O; I_2_ + H_2_O ⇌ HOI + I^−^ + H^+^	Produces I_2_, which in aqueous systems is partly converted to HOI. HOI is short-lived but is the inorganic iodine species that most readily partitions into the gas phase, enabling transfer of iodine from liquid to air.
A2: Organification (MIT, DIT formation)	HOI + Tyr → MIT + H_2_O; MIT + HOI → DIT + H_2_O	Fixes iodine in organic form; radiolysis and metabolism may release volatile organoiodine species.
A3: Radiolytic oxidation	H_2_O → •OH + •H; •OH + I^−^ → •I + OH^−^	β^−^-radiation from ^131^I induces radiolysis of water, forming •OH and •I radicals that drive non-enzymatic oxidation and initiate methylation pathways leading to CH_3_I.
A4: Radiolytic methylation	•I + •CH_3_ → CH_3_I	^131^I β^−^-radiation induces radiolysis of water and organic molecules, generating •I and •CH_3_ radicals; their recombination forms CH_3_I, explaining the predominantly organic fraction of exhaled iodine.
(**B**)		
B1: Radiolytic oxidation in coolant	Radiolytic oxidation of iodide in coolant water (γ-induced)	Ionizing radiation in the coolant—dominated by γ-radiation from the fuel—induces radiolysis of water, generating •OH radicals that oxidize I^−^ to I_2_/HOI.
B2: Hydrolysis/disproportionation	I_2_ + H_2_O ⇌ HOI + I^−^ + H^+^	For the gas-phase relevance of HOI, see explanation in Medical Context (A1).
B3: Metal iodide formation	I_2_ + reactor metal surfaces → non-volatile metal–iodine species	Metal surfaces (steel, alloys) form non-volatile iodine species that act as sinks and reduce airborne inorganic iodine during operation and especially during shutdown/inspection.
B4: Radiolytic/thermal methylation → CH_3_I	•I + •CH_3_ → CH_3_I	CH_3_I can form by radiolytic or thermal methylation of iodine in the reactor environment. Under normal operating conditions, formation is very limited, but CH_3_I becomes relevant in high-radiation or off-normal situations because it is poorly retained by standard charcoal filters.
(**C**). Reprocessing/Mo-99 Production		
C1: Nitrate-driven oxidation	2 I^−^ + 2 NO_3_^−^ + 4 H^+^ → I_2_ + 2 NO_2_ + 2 H_2_O I_2_ + H_2_O ⇌ HOI + I^−^ + H^+^	Concentrated HNO_3_ used in Mo-99 processing oxidizes iodide to I_2_. In the acidic aqueous phase, a fraction of I_2_ undergoes hydrolysis to HOI, and both species can transfer into the off-gas during acid handling.
C2: HNO_2_/NO_2_ redox cycling (sustained I_2_ release)	2 I^−^ + 2 HNO_2_ + 2 H^+^ → I_2_ + 2 NO + 2 H_2_O	Radiolytic formation of nitrous acid (HNO_2_) regenerates oxidizing equivalents, producing sustained I_2_ release during dissolution.
C3: Radiolytic/thermal methylation	•CH_3_ + •I → CH_3_I	Residual organic compounds in Mo-99 processing (e.g., solvents, extractants, degradation products) can undergo radiolytic or thermal decomposition, forming methyl radicals that react with iodine to produce CH_3_I. Although usually minor, CH_3_I is relevant because it is the most volatile iodine species and is poorly captured by standard charcoal filters
(**D**)		
D1: Thermal decomposition/oxidation of CsI → I_2_ (gas)	I^−^ → I_2_ (thermal)	In reactor fuel, iodine fission products are predominantly incorporated as cesium iodide (CsI, largely with stable ^133^Cs). During high-temperature fuel degradation, CsI is released and decomposes or oxidizes on metal and oxide surfaces to form gaseous I_2_.
D2: Radiolytic formation of H_2_O_2_ and oxidation of iodide	2 I^−^ + 2 H^+^ + H_2_O_2_ → I_2_(g) + 2 H_2_O I^−^ + H_2_O_2_ → HOI(g) + OH^−^	Under severe accident conditions, intense γ-radiation splits water (H_2_O → H_2_O_2_, H_2_, e^−^_a_q), forming hydrogen peroxide as a stable radiolysis product. H_2_O_2_ then oxidizes iodide to volatile I_2_ and HOI, which enter the steam phase.
D3: Gas-phase photolysis of I_2_	I_2_ →(hν) 2 I•	Once released, I_2_ undergoes rapid photolysis to reactive I-atoms, initiating atmospheric oxidation to iodine oxides. Relevant for atmospheric transformation, not for source-term generation.
D4: Gas-phase oxidation to higher iodine oxides → aerosol nuclei	I• + O_3_ → IO• → … → I_2_O_5_(s) (s)	Schematic pathway: iodine radicals (I•) formed from I_2_ react with ozone and oxygen in the gas phase to yield higher iodine oxides (e.g., I_2_O_5_), which nucleate or condense as particulate aerosols.
D5: M (metal vapor) + I_2_ → MI_x_ (M = Cs, Zr, Ag; x = 1–4 depending on oxidation state and temperature)	M + (x/2) I_2_ → MI_x_ (x = 1–4) e.g., Cs + ½ I_2_ → CsI Zr + 2 I_2_ → ZrI_4_ Ag + ½ I_2_ → AgI	During severe core degradation, temperatures exceed the volatilization thresholds of cesium, zirconium, and structural silver. In this high-temperature gas phase, vaporized metals react rapidly with molecular iodine to form metal iodides (e.g., CsI, ZrI_4_, AgI), which subsequently condense as fine aerosols during cooling.
D6: Formation of particulate iodine aerosols (condensation and droplets)	MI_x_(g) → MI_x_(s) (0.1–1 µm) I^−^/CsI (in aqueous film) → aerosol droplets	Cooling of iodine-bearing gases leads to condensation of volatile metal iodides (CsI, ZrI_4_, AgI) into submicron solid aerosols. Additionally, boiling or disturbance of contaminated liquid films can generate iodide-containing droplets. Both pathways produce inhalable particulate iodine during late accident phases.
D7: Coating interactions: adsorption, reduction, and γ-radiolysis-driven CH_3_I formation	I_2_ + coating surface ⇌ I_2_ (surface) → I^−^/R–I CH_3_• (from γ-radiolysis of organic coatings) + I_2_/HOI → CH_3_I	Containment coatings (epoxy/alkyd/amine) initially adsorb I_2_ and reduce it to iodide or bound organoiodine. Under γ-irradiation, the organic matrix undergoes radiolysis, producing methyl radicals (CH_3_•) that react with I_2_ or HOI to form volatile CH_3_I.
D8: Fire and soot environment: radical-driven CH_3_I formation and carbonaceous iodine uptake	(organic material → combustion/γ → CH_3_•, R•) CH_3_• + I_2_/HOI → CH_3_I I_2_/HOI + soot → I–C(surface)	Combustion and γ-irradiation of organic materials generate abundant methyl and organic radicals that react with I_2_ or HOI to form volatile CH_3_I. Simultaneously, soot and other carbonaceous surfaces adsorb I_2_ and HOI, producing particle-bound iodine that can be transported as fine aerosols.

Notes: (A). MIT, monoiodotyrosine; DIT, diiodotyrosine; Tyr, tyrosine. Radical species (•OH, •I, •CH_3_) denote radiolytically generated intermediates. Reactions are shown in simplified form to illustrate dominant transformation pathways of radioiodine, predominantly I-131, under physiological and radiolytic conditions. (B). Radiolysis refers to ionizing radiation–induced decomposition of water in the reactor coolant, generating reactive species such as •OH and •H. HOI denotes hypoiodous acid formed by hydrolysis of I_2_ in aqueous systems. Metal–iodine interactions summarize the formation of non-volatile metal iodides on reactor surfaces (e.g., steel or alloy components), which act as sinks for inorganic iodine. Reaction schemes are shown in simplified form to highlight dominant chemical transformation pathways of radioiodine (predominantly I-131) under normal and off-normal reactor operating conditions. (C). HNO_3_ denotes nitric acid used in Mo-99 production and spent-fuel reprocessing, providing strongly oxidizing conditions that convert iodide to molecular iodine (I_2_). NOx refers to nitrogen oxides generated during radiolysis and redox cycling, including nitrous acid (HNO_2_), which sustain oxidizing equivalents and promote continued iodine release. HOI (hypoiodous acid) is formed by hydrolysis of I_2_ in acidic aqueous phases and facilitates transfer of iodine into the off-gas. Radiolytic and thermal degradation of organic process residues (e.g., solvents, extractants, degradation products) generates methyl radicals (•CH_3_), enabling formation of volatile organoiodine species such as CH_3_I. Reaction schemes are simplified to highlight dominant chemical transformation pathways of radioiodine (predominantly I-131) under reprocessing conditions. (D). M denotes reactor structural metals (e.g., Cs, Zr, Ag) and X denotes halogen stoichiometry (X = 1–4). HOI is hypoiodous acid; H_2_O_2_ denotes hydrogen peroxide formed under intense radiolysis. Radical species (e.g., •I, •OH, •CH_3_) indicate radiolytically generated intermediates; (hν) denotes photolysis. “Surface” represents reactive interactions with oxide layers or containment coatings. Reaction schemes are simplified to emphasize dominant high-temperature, radiolytic, and aerosol-forming pathways of radioiodine (predominantly I-131) under severe-accident conditions.

**Table 3:** Core Intersection Points Across All Four Radioiodine Release Scenarios.

Reaction/Mechanism	Nuclear Medicine (RIT, Exhalation)	Routine Reactor Operation	Reprocessing/Mo-99 Production (Incl. Shutdown/Inspection)	Severe Reactor Accidents (LOCA, Fire, Loss of Containment)
Peroxidase-driven oxidation (H_2_O_2_/I_2_/HOI)	✓ (TPO/LPO)	–	–	–
Radiolytic oxidation (•OH, •I; H_2_O_2_ formation)	✓ (β^−^ from I-131)	✓ (γ-fields in coolant)	✓	✓
Radiolytic methylation/CH_3_• transfer	✓ (oxidative stress)	–	✓ (organic solvents, diluents)	✓ (fire/soot radicals)
Hydrolysis/disproportionation (I_2_ ⇌ HOI + I^−^ + H^+^)	✓	✓	✓	✓
Organification/iodination of organic matter	✓ (thyroglobulin/tissue)	–	✓ (organic phase, solvents)	✓ (coatings, soot organics)
Formation of metal iodides (CsI, ZrI_4_, AgI)	–	✓ (surface corrosion)	✓ (hot dissolver metals)	✓ (high-T vapor phase)
Aerosol formation (condensed MI_x_ or liquid droplets)	–	–	✓ (acid systems, boiling/flashing)	✓ (MI_x_ condensation; droplet aerosolization)
Coating interactions (adsorption, CH_3_I formation)	–	–	–	✓ (epoxy/alkyd/amine under γ)
Fire/soot chemistry and carbonaceous iodine uptake	–	–	–	✓ (CH_3_• formation + soot adsorption)

Notes: TPO, thyroid peroxidase; LPO, lactoperoxidase; HOI, hypoiodous acid; CH_3_I, methyl iodide; I_2_, molecular iodine; MI_x_, generic metal iodides (e.g., CsI, ZrI_4_, AgI); R•/CH_3_•, organic and methyl radicals produced by radiolysis or combustion. A check mark (✓) indicates that the respective reaction or mechanism is relevant or observed in the given scenario.

**Table 4:** I-131: Relationship between Chemical Form and Effective Dose Coefficients.

Route of Intake	Physicochemical Form/Absorption Type	Members of the Public (ICRP 119, Annex G & F) [Sv Bq^−1^]	Workers (ICRP 119, Annex B) [Sv Bq^−1^]	Comment
Inhalation	Gas, [^131^I]CH_3_I (methyl iodide)	≈7.6 × 10^−9^ (Type F equivalent)	1.5 × 10^−8^	Volatile organic iodine; rapid pulmonary absorption; explicitly listed in Annex B.
Inhalation	Gas, [^131^I]I_2_ (molecular iodine)	≈7.6 × 10^−9^ (Type F equivalent)	2.0 × 10^−8^	Reactive molecular iodine vapor; complete absorption assumed; Annex B.
Inhalation	Aerosol, Type F (fast soluble)	7.6 × 10^−9^	–	Fully soluble iodide or iodate particles; immediate systemic availability.
Inhalation	Aerosol, Type M (moderately soluble)	1.2 × 10^−8^	–	Partially soluble particles; delayed absorption via macrophage clearance.
Inhalation	Aerosol, Type S (slow soluble)	9.4 × 10^−9^	–	Poorly soluble metal iodides (e.g., [^131^I]AgI); long alveolar retention; slow systemic uptake.
Ingestion	[^131^I]I^−^/[^131^I]I_2_ (soluble oral forms)	1.1 × 10^−8^–1.2 × 10^−8^	–	Gastrointestinal absorption of inorganic or molecular iodine; nearly complete bioavailability.

Data source: International Commission on Radiological Protection (ICRP) Publication 119, Compendium of Dose Coefficients based on ICRP Publication 60 [12]. Values represent committed effective dose coefficients for inhalation and ingestion of ^131^I under reference biokinetic assumptions. Worker data (Annex B) apply to occupational exposure to reactive iodine vapors, while public data (Annexes F–H) are used for environmental and patient-related scenarios. Minor differences (<20%) between worker and public models fall within the inherent uncertainty of biokinetic parameters. Volatile iodine species such as [^131^I]CH_3_I and [^131^I]I_2_ show dose coefficients comparable to Type F aerosols, reflecting their rapid pulmonary absorption and systemic distribution, whereas poorly soluble metal iodides (Type S) exhibit slower dissolution, leading to prolonged alveolar retention and lower immediate thyroid dose.

## Data Availability

This review did not generate or analyze new primary data. Therefore, data sharing is not applicable.
